# Dual Functionalized *Lactococcus lactis* Shows Tumor Antigen Targeting and Cytokine Binding *in Vitro*

**DOI:** 10.3389/fbioe.2022.822823

**Published:** 2022-01-26

**Authors:** Abida Zahirović, Tina Vida Plavec, Aleš Berlec

**Affiliations:** ^1^ Department of Biotechnology, Jožef Stefan Institute, Ljubljana, Slovenia; ^2^ Faculty of Pharmacy, University of Ljubljana, Ljubljana, Slovenia

**Keywords:** cancer, probiotics, *Lactococcus lactis*, targeting, cytokines, IL-6, IL-8

## Abstract

Pro-inflammatory cytokines play an important role in the development and progression of colorectal cancer (CRC). Tumor-targeting bacteria that can capture pro-inflammatory cytokines in the tumor microenvironment and thus block their tumor-promoting effects might provide clinical benefits in inflammation-associated CRC. The aim of this study was to develop bacteria with dual functionality for selective delivery of cytokine-binding proteins to the tumor by targeting specific receptors on cancer cells. We engineered a model lactic acid bacterium, *Lactococcus lactis*, to co-display on its surface a protein ligand for tumor antigens (EpCAM-binding affitin; HER2-binding affibody) and a ligand for pro-inflammatory cytokines (IL-8-binding evasin; IL-6-binding affibody). Genes that encoded protein binders were cloned into a lactococcal dual promoter plasmid, and protein co-expression was confirmed by Western blotting. To assess the removal of IL-8 and IL-6 by the engineered bacteria, we established inflammatory cell models by stimulating cytokine secretion in human colon adenocarcinoma cells (Caco-2; HT-29) and monocyte-like cells (THP-1; U-937). The engineered *L. lactis* removed considerable amounts of IL-8 from the supernatant of Caco-2 and HT-29 cells, and depleted IL-6 from the supernatant of THP-1 and U-937 cells as determined by ELISA. The tumor targeting properties of the engineered bacteria were evaluated in human embryonic kidney epithelial cells HEK293 transfected to overexpress EpCAM or HER2 receptors. Fluorescence microscopy revealed that the engineered *L. lactis* specifically adhered to transfected HEK293 cells, where the EpCAM-targeting bacteria exhibited greater adhesion efficiency than the HER2-targeting bacteria. These results confirm the concept that *L. lactis* can be efficiently modified to display two proteins simultaneously on their surface: a tumor antigen binder and a cytokine binder. Both proteins remain biologically active and provide the bacteria with tumor antigen targeting and cytokine binding ability.

## Introduction

Chronic, unresolved inflammation has been increasingly recognized as a key factor in the pathogenesis of many types of cancers, including liver, pancreatic, gastric, and colorectal cancers (CRC) (Terzic et al., 2010). CRC is the third most common cancer worldwide, and the second leading cause of cancer-related deaths (Sung et al., 2021). Studies have provided strong evidence that the pro-inflammatory cytokines interleukin (IL)-6 and IL-8 (CXCL-8) have critical roles in the inflammatory processes associated with CRC (Baier et al., 2005; West et al., 2015). IL-6 has been shown to promote tumor cell growth, invasion, and migration (Becker et al., 2004; Zeng et al., 2017), while IL-8 increases proliferation, angiogenesis, and migration of malignant cells toward blood vessels, thus leading to tumor dissemination (Ning et al., 2011). Clinical data show that patients with stage IV CRC have more than 10 times higher serum level of IL-8 (1,089 pg/ml) than healthy individuals (79 pg/ml) (Lee et al., 2012). Of the pro-inflammatory cytokines involved in CRC carcinogenesis, IL-6 shows the greatest increase in patients with CRC compared to healthy controls, particularly in metastatic disease (Chung and Chang, 2003). Elevated IL-6 correlates with a bad patient prognosis and poor clinical outcome of CRC. Furthermore, pro-inflammatory cytokines have been shown to reduce the responses to cancer immunotherapy by inducing immunosuppression in the tumor microenvironment (Albini and Sporn, 2007).

A growing body of data on the critical role of pro-inflammatory cytokines at various stages of CRC development suggests that they represent new molecular targets in the treatment of CRC. Indeed, recent studies have demonstrated inhibitory effects of cytokine signaling blockers on cancer development and metastasis (Ning and Lenz, 2012; Johnson et al., 2018). These therapeutics will probably require combinatorial approaches, and might therefore serve as adjuvant therapies following standard treatment with radiotherapy, chemotherapy, or immune checkpoint inhibitors (Wattenberg and Beatty, 2020).

When considered for cancer treatment, local delivery of cytokine-binding proteins to the tumor is necessary to avoid systemic cytokine blockade, and to thus circumvent the side effects associated with down-regulation of the normal immune response. Site-specific drug delivery ensures maximal concentrations of therapeutic agents at the local site of action without negative side effects in healthy tissues. Sustained high levels of cytokine-binding proteins in tumor tissue can be achieved by targeting their delivery to the tumor using bacteria as the delivery vehicle. One of the main advantages of using bacteria for cancer treatments is their natural propensity for the central hypoxic regions of a tumor. This is a prominent feature of anerobic or facultative anaerobic bacterial pathogens such as *Salmonella*, *Listeria*, and *Clostridium*. However, even when these bacteria are used in an attenuated form, there is a risk of reversion to a virulent state (Toso et al., 2002).

*Lactococcus lactis* (*L. lactis*), a model lactic acid bacterium, raises no safety concerns and is considered a suitable host strain for development of biotherapeutics. *L. lactis* is particularly advantageous for intestinal drug delivery as it can survive the passage through the gastrointestinal tract (Plavec and Berlec, 2019; Plavec and Berlec, 2020). *L. lactis* can serve as a protein producer and a delivery vehicle at the same time, and it has previously been engineered for gastrointestinal delivery of various proteins, including viral antigens (Berlec et al., 2013), allergens (Zahirović and Lunder, 2018), immunomodulatory cytokines (Steidler et al., 2003) and proteins with affinity for pro-inflammatory cytokines (Ravnikar et al., 2010; Berlec et al., 2017; Kosler et al., 2017; Škrlec et al., 2018; Plavec et al., 2019) and chemokines (Škrlec et al., 2017). Moreover, alternative methods to genetic modification of bacteria have been introduced, including heterologous protein expression (Zadravec et al., 2015) and containment strategies (Steidler et al., 2003). Some applications were also aimed at the treatment of cancer (primarily CRC); these include *L. lactis* engineered to deliver antioxidant enzyme catalase (Del Carmen et al., 2017) or pro-apoptotic peptide kisspeptin (Zhang et al., 2016).

The wild-type lactic acid bacteria have shown protective effects against CRC through modulation of the gut microbiota, neutralization of carcinogens, and/or production of short-chain fatty acids (Uccello et al., 2012). These inherent anticancer effects can be enhanced by displaying cytokine-binding proteins on the bacterial surface, which will result in the capture of pro-inflammatory cytokines by the bacteria, and neutralization of their detrimental pro-tumorigenic effects in the tumor milieu. Furthermore, expression of specific proteins directed towards tumor antigens on the bacterial surface can enhance their selectivity for tumors (Duong et al., 2019). This was successfully achieved for attenuated *Salmonella typhimurium* by coating the bacterial surface with the RGD peptide that is directed against αvβ3-integrin (Park et al., 2016), or with a single-domain antibody directed against the B-lymphocyte antigen CD20 (Massa et al., 2013).

We have recently developed *L. lactis* strains that display small protein binders of IL-8 (Škrlec et al., 2017) and IL-6 (Zahirović et al., submitted) on their surface, and have demonstrated their binding to cytokines *in vitro*. Both proteins that were displayed on *L. lactis*, IL-8-binding evasin (Deruaz et al., 2008) and IL-6-binding affibody (Yu et al., 2014), are non-immunoglobulin binders and have high specificity and affinity for their target cytokines. We reasoned that the co-display of cytokine-binding proteins with ligands for tumor antigens can facilitate the specific interactions of the bacteria with the cancer cells, which will increase the accumulation and retention of the bacteria in the tumor tissue, and thus enable the targeted delivery of the cytokine-binding proteins to the tumors.

For tumor antigen-targeting we used epithelial cell adhesion molecule (EpCAM, CD326) (Das et al., 2015) and human epidermal growth factor receptor 2 (HER2, CD340) (Siena et al., 2018); both are up-regulated in CRC, but not in healthy cells, and therefore represent CRC markers. Small non-immunoglobulin scaffold proteins, EpCAM-binding affitin (Kalichuk et al., 2018), and HER2-binding affibody (Feldwisch et al., 2010), that possess high specificity and picomolar affinity for their receptors served as the targeting ligands. *L. lactis* bacteria that displayed these tumor antigen ligands on their surface were recently shown to specifically bind to tumor surface antigens (Plavec et al., 2021b). Lectin-displaying *L. lactis* that target carbohydrate receptors on cancer cells were also developed (Plavec et al., 2021c).

In the present study, we sought to develop bacteria for targeted delivery of cytokine-binding proteins to tumors. We engineered *L. lactis* to display a combination of a tumor antigen-recognizing protein (EpCAM-binding affitin AFFI or HER2-binding affibody ZHER) and a cytokine binding protein (IL-8–binding evasin EVA or IL-6–binding affibody ZIL) on their surface. The engineered bacteria were tested for their ability to remove IL-8 and IL-6 from the supernatant of cancer cells and to bind tumor antigens EpCAM or HER2 on HEK293 cells.

## Materials and Methods

### Bacterial Strains, Media, and Growth Conditions

The bacterial strains used in this study are shown in Table 1. *Lactococcus lactis* NZ9000 was grown at 30°C in M17 medium (MilliporeSigma, Burlington, MA, United States) supplemented with 0.5% glucose (Fluka AG, Buchs, Switzerland) (GM-17 medium) without agitation, or in the same medium solidified with 1.5% agar (Formedium, Hunstanton, United Kingdom). To maintain selection pressure on *L. lactis* transformants, 10 μg/ml chloramphenicol (Sigma-Aldrich, St. Louis, MO, United States) was added to the growth medium. Biliverdin HCl (15.5 μg/ml; Sigma-Aldrich) was added for expression of infrared fluorescent protein (IRFP).

**TABLE 1:** The strains, plasmids and primers used in this study.

Strain, plasmid or primer	Relevant features or sequence	Reference
Strain		
*L. lactis* NZ9000	MG1363 *nisRK* Δ*pepN*	NIZO
Plasmids		
pNZ8148	pSH71 derivative, P_*nisA*_ *,* Cm^r^, nisin-controlled expression	de Ruyter et al. (1996), Mierau and Kleerebezem. (2005)
pNZ-AFFI-IRFP	pNZ8148-containing gene fusion of *sp*_Usp45_, *affi* and *acmA3b* on MCS1 and *irfp* gene on MCS2	Plavec et al. (2021b)
pNZ-ZHER-IRFP	pNZ8148-containing gene fusion of *sp*_Usp45_, *flag-zher* and *acmA3b* on MCS1 and *irfp* gene on MCS2	Plavec et al. (2021b)
pNZ-IRFP	pNZ8148-containing *irfp* gene	Berlec et al. (2015)
pSD-EVA	pNZ8148-containing gene fusion of *sp*_Usp45_, *evasin-3* and *acmA3b*	Škrlec et al. (2017)
pSD-ZIL	pNZ8148-containing gene fusion of *sp*_Usp45_, *zil* and *acmA3b*	Zahirović et al., submitted
pNZ-AFFI-EVA-IRFP	pNBBX-containing AFFI, EVA and IRFP cassettes	Plavec et al. (2021a)
pNZ-AFFI-ZIL-IRFP	pNBBX-containing AFFI, ZIL and IRFP cassettes	Plavec et al. (2021a)
pNZ-ZHER-EVA-IRFP	pNBBX-containing ZHER, EVA and IRFP cassettes	Plavec et al. (2021a)
pNZ-ZHER-ZIL-IRFP	pNBBX-containing ZHER, ZIL and IRFP cassettes	Plavec et al. (2021a)
pNZ-AFFI-EVA	pNZ8148-containing gene fusion of *sp*_Usp45_, *affi* and *acmA3b* on MCS1 and gene fusion of *sp*_Usp45_, *eva* and *acmA3b* on MCS2	This study
pNZ-AFFI-ZIL	pNZ8148-containing gene fusion of *sp*_Usp45_, *affi* and *acmA3b* on MCS1 and gene fusion of *sp*_Usp45_, *zil* and *acmA3b* on MCS2	This study
pNZ-ZHER-EVA	pNZ8148-containing gene fusion of *sp*_Usp45_, *flag-zher* and *acmA3b* on MCS1 and gene fusion of *sp*_Usp45_, *eva* and *acmA3b* on MCS2	This study
pNZ-ZHER-ZIL	pNZ8148-containing gene fusion of *sp*_Usp45_, *flag-zher* and *acmA3b* on MCS1 and gene fusion of *sp*_Usp45_, *zil* and *acmA3b* on MCS2	This study
Primers		
USP-F-Nde2	TTA​TTT​CAT​ATG​GCT​AAA​AAA​AAG​ATT​ATC​TCA​G	Škrlec et al. (2017)
A3b-R-Xho2	TTA​TTT​CTC​GAG​TTA​TTT​TAT​TCG​TAG​ATA​CTG​ACC	Škrlec et al. (2017)

NIZO, www.nizo.com; MCS, multiple cloning site.

### Construction of Dual Protein Expression Plasmids

**FIGURE 1:**
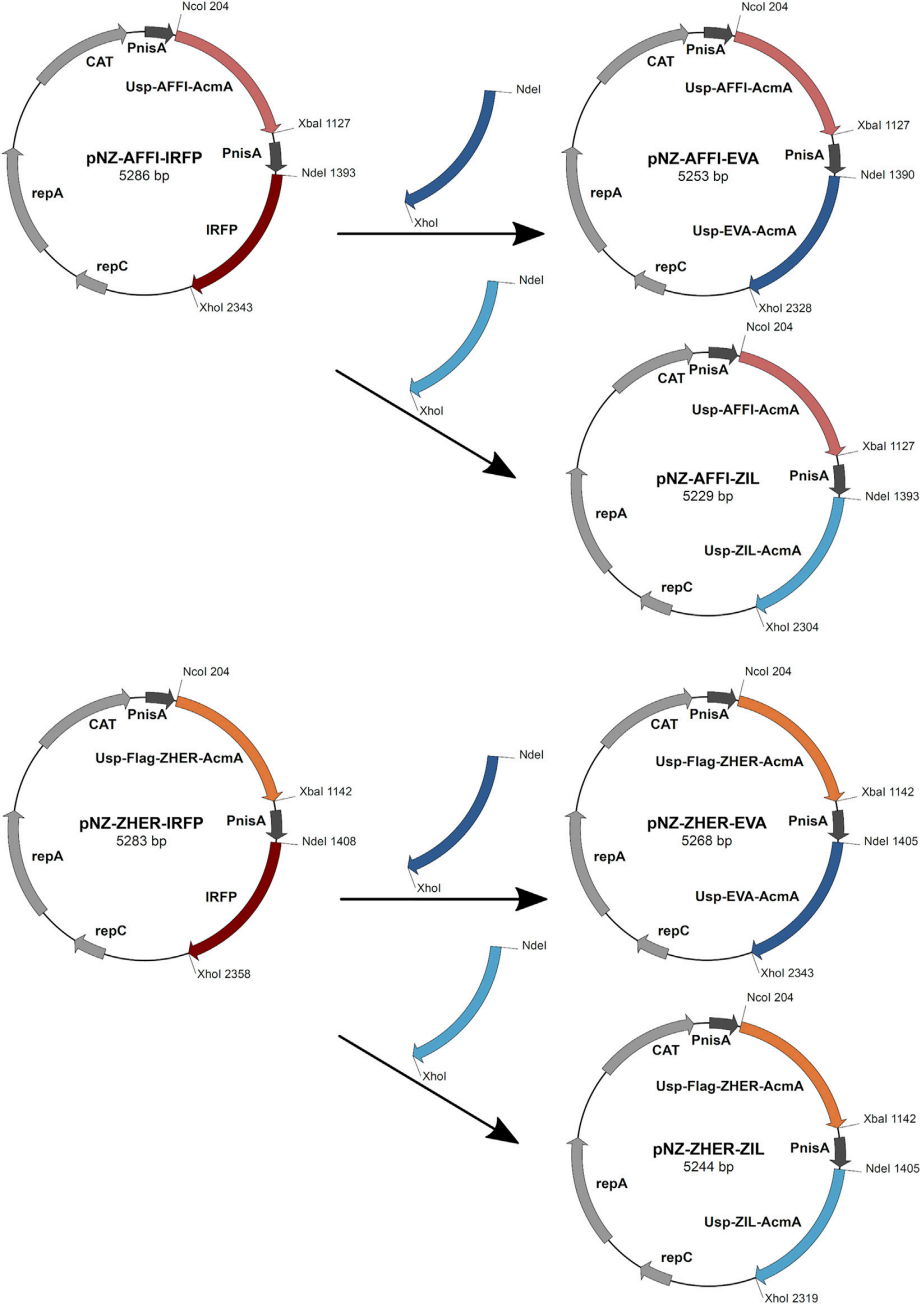
The scheme of the construction of dual expression plasmids. For construction of dual plasmids for surface display of tumor antigen binders and cytokine binders, we used previously prepared pNZDual plasmids which already contained expression cassette with the tumor antigen binder gene (ZHER or AFFI) in multiple cloning site 1 and IRFP in multiple cloning site 2. IRFP was substituted by the expression cassette containing the cytokine binder gene (EVA or ZIL). Thus, tumor antigen binder was paired with a cytokine binder, resulting in four dual plasmids pNZ-AFFI-EVA, pNZ-AFFI-ZIL, pNZ-ZHER-EVA, and pNZ-ZHER-ZIL. In each expression cassette, the binder gene was fused with the gene for Usp45 secretion signal and the gene for peptidoglycan binding domain of the AcmA protein, to enable surface display of the binder following its expression. AFFI, gene encoding EpCAM-binding affitin (183 bp); ZHER, gene encoding HER2-binding affibody (174 bp); EVA, gene encoding IL-8-binding evasin-3 (243 bp); ZIL, gene encoding IL-6-binding affibody (174 bp); Usp, gene encoding Usp45 secretion signal peptide (84 bp); AcmA, gene encoding the C-terminal portion of the AcmA surface anchoring protein containing three LysM repeats (642 bp); FLAG, epitope tag sequence; PnisA, nisin promoter.

Restriction enzymes and T4 DNA ligase were from New England Biolabs (Ipswich, MA, United States). PCR amplifications were performed with Taq polymerase (New England Biolabs), according to the manufacturer protocols. Plasmid DNA was isolated (NucleoSpin plasmid; Macherey-Nagel, Düren, Germany), with addition of lysozyme (Sigma-Aldrich). Electroporation of *L. lactis* was performed according to (Holo and Nes, 1995) (Gene Pulser II apparatus; BioRad, Hercules, CA, United States). Nucleotide sequencing was performed by Eurofins Genomics (Ebersberg, Germany). Primers (Integrated DNA Technologies, Leuven, Belgium) and plasmids are listed in Table 1. Protein binders were introduced into the pNZDual plasmid using restriction enzyme-based cloning. Plasmid pNZDual contains two multiple cloning sites (MCS), each preceded by a nisin promoter, which enables expression of two proteins in *L. lactis* (Berlec et al., 2018). To construct dual expression plasmids that encode tumor antigen binder along with cytokine binder, we used pNZDual plasmids which already contained expression cassette with the tumor antigen binder gene (*affi* or *zher*) in MCS 1 and IRFP in MCS 2 [prepared previously in (Plavec et al., 2021b)], referred to as pNZ-AFFI-IRFP and pNZ-ZHER-IRFP, respectively. We replaced IRFP in the MCS 2 with the expression cassette containing the cytokine binder gene (*eva* or *zil*) *via* NdeI/XhoI restriction sites. Thus, tumor antigen binder was paired with a cytokine binder, resulting in four dual plasmids pNZ-AFFI-EVA, pNZ-AFFI-ZIL, pNZ-ZHER-EVA, and pNZ-ZHER-ZIL (Table 1). A construction of dual protein expression plasmids is depicted in Figure 1. In each expression cassette, the binder gene was fused with the gene for Usp45 secretion signal and the gene for peptidoglycan binding domain of the AcmA protein, to enable its release into the growth medium and subsequent surface anchoring. The expression cassette containing cytokine binder gene *eva* or *zil* was amplified from pSD-EVA (Škrlec et al., 2017) or pSD-ZIL plasmid (Zahirović et al., submitted), respectively. For generation of pSD-ZIL plasmid, *zil* gene was back-translated and codon-optimized for use in *L. lactis* based on the amino acid sequence of IL-6-binding affibody ZIL-6_13 (Yu et al., 2014). *Zil* gene was cloned into a lactococcal plasmid pSDBA3b in the same way as evasin 3 following the protocol described in Škrlec et al. (2017). For visualization of engineered bacteria, IRFP-encoding gene was subsequently inserted into the plasmid along with the protein binders, using BglBrick cloning, which allowed easier assembly of multiple gene cassettes (Plavec et al., 2021a).

### Expression of Fusion Proteins in *L. lactis*

Overnight cultures of *L. lactis* harboring constructed plasmids were diluted (1:50) in 10 ml fresh GM-17 medium, and grown to an optical density (A_600_) of 0.50–0.80. Expression of the fusion proteins was induced by addition of 25 ng/ml nisin (Fluka AG) (de Ruyter et al., 1996; Mierau and Kleerebezem, 2005). After a 3 h incubation, 1 ml culture was stored at 4°C for flow cytometric analysis, and the remaining cell culture was centrifuged at 5,000× *g* for 10 min. The cell pellet was resuspended in 400 µl phosphate-buffered saline (PBS; 10 mM Na_2_HPO4, 1.8 mM KH_2_PO_4_, 137 mM NaCl, and 2.7 mM KCl; pH 7.4) and stored at −20°C for SDS-PAGE analysis, or resuspended in different volumes of PBS for analysis of cytokine binding by ELISA.

### SDS-PAGE and Western Blotting

SDS-PAGE was performed with a Mini-Protean II apparatus (BioRad). Samples were thawed in an ice bath, briefly sonicated (UPS200S; Hielscher, Teltow, Germany), mixed with 2× Laemmli sample buffer and dithiothreitol, and denatured by heating at 100°C before loading. Pre-stained standards (Page Ruler Plus; Thermo Fisher Scientific, Waltham, MA, United States) were used for molecular weight comparisons. Proteins were transferred onto nitrocellulose membrane (GE Healthcare Life Sciences, Marlborough, MA, United States) using semi-dry transfer at 100 V for 90 min. Membranes were blocked with 5% non-fat dried milk in TBS (50 mM Tris-HCl, 150 mM NaCl; pH 7.5) with 0.05% Tween-20 (TBST) and incubated overnight at 4°C in blocking buffer with target proteins. For detection of protein binders, the following target proteins were used: for AFFI, EpCAM/TROP1 Fc chimera (R&D Systems, Minneapolis, MN, United States); for ZHER, ErbB2/Her2 Fc chimera (R&D Systems); for EVA, human IL-8 conjugated with Fc region (Sinobiological, China); and for ZIL, biotinylated human IL-6 (ImmunoTools, Friesoythe, Germany). Proteins conjugated to Fc region were subsequently incubated with goat anti-human Fc antibody (1:1,000, Jackson ImmunoResearch, West Grove, PA, United States), whereas IL-6 conjugated to biotin was incubated with mouse anti-biotin antibody (1:1,000, Abcam, Waltham, MA, United States). After three washes with TBST, the membranes were incubated with horseradish peroxidase (HRP)-conjugated secondary antibodies diluted 1:5,000 in blocking buffer, for 1.5 h. The secondary antibodies used were HRP-conjugated anti-mouse (1:5,000; Jackson ImmunoResearch) and HRP-conjugated anti-goat (1:5,000; Jackson ImmunoResearch). Following three washes with TBST, the membranes were incubated with a chemiluminescent reagent (Clarity Western ECL Substrate; Bio-Rad). Images were acquired using an imaging system (ChemiDoc MP; BioRad).

### ELISA for Determination of Cytokine Binding by *L. lactis*

The cytokine concentrations were determined using commercially available ELISA kits (Mabtech, Nacka Strand, Sweden), according to the manufacturer recommendations. Here, 96-well plates (Maxisorp Nunc; Thermo Fisher Scientific) were coated with cytokine-binding antibodies at 4°C overnight. The wells were washed five times with 200 μl PBS containing 0.05% Tween-20 (wash buffer) and blocked for 1 h at room temperature with PBS with 0.05% Tween and 0.1% bovine serum albumin (incubation buffer). *L. lactis* cells at 6 × 10^9^ colony forming units (CFU)/ml were centrifuged (5,000 × *g*, 5 min, 4°C) and resuspended in 250 µl incubation buffer spiked with 300 pg/ml cytokine standards (Mabtech). The optical density of bacterial suspension at 600 nm (OD_600_) was used to calculate the number of *L. lactis* per ml using a factor determined earlier by serial dilutions (1 OD_600_ = 1 ×10^9^ *L. lactis*/ml). Following 2 h at room temperature with gentle shaking, the bacterial cells were removed by centrifugation (7,500× *g*, 7 min, room temperature), and 200 μl cell-free bacterial supernatant was added to the wells and incubated for 2 h at room temperature. The remaining cytokine levels in the supernatant were determined using a calibration curves, that were generated by addition of different concentrations (0–1,200 pg/ml) of recombinant cytokine standards from the ELISA kit (Mabtech). The standards were incubated in the same buffer and under the same conditions as the samples, to ensure equal matrix effect. Following incubation and washing of the wells, 100 μl biotinylated monoclonal antibodies against the cytokines were added in incubation buffer at their recommended concentrations, and incubated at room temperature for 1 h. Following washing, 100 μ streptavidin-HRP (diluted 1:1,000 in incubation buffer) was added into the wells and incubated for 1 h at room temperature. The wells were washed again, and 50 μl 3,3′,5,5′-tetramethylbenzidine substrate (Sigma-Aldrich) was added. The reaction was terminated after 20 min by addition of 50 μl 2 M sulfuric acid. Absorbances were read at 450 nm using a microplate reader (Infinite M1000; Tecan, Grödig, Austria), with wavelength correction at 650 nm. The binding is given as the proportions (%) of the cytokine removed from the solution by the bacteria.

### Cell Lines and Culturing

Human embryonic kidney epithelial HEK293 cells (CRL-1573; American Type Culture Collection [ATCC], Manassas, Virginia, United States) and human colon adenocarcinoma Caco-2 cells (HTB-37; ATCC) were cultured in Dulbecco’s modified Eagle’s medium with high glucose and GlutaMAX (Gibco, Thermo Fisher Scientific). Human colon adenocarcinoma HT29 epithelial intestinal cells (HTB-38; ATCC) were maintained in McCoy’s medium with 1.5 mM L-glutamine and 2,200 mg/L sodium bicarbonate (ATCC). Human monocytic cell lines THP-1 (TIB-202; ATCC) and U-937 (CRL-1593.2; ATCC) were cultured in RPMI1640 medium (Gibco). Each medium was supplemented with 10% (v/v) fetal bovine serum (Gibco), 100 U/mL penicillin, and 100 μg/ml streptomycin (Gibco). For Caco-2 cell culturing, 25 mM HEPES (Sigma-Aldrich) and 1% Eagle’s minimum essential medium nonessential amino acids solution (Sigma-Aldrich) were added. Unless otherwise stated, the cells were seeded in 24-well tissue culture plates (Corning, Thermo Fisher Scientific) at 1 ×10^5^ or 3 ×10^5^ cells/well and incubated at 37°C in a humidified atmosphere containing 5% CO_2_.

### Cell Differentiation, Stimulation of Cytokine Production, and Incubation With Bacteria

THP-1 and U-937 cells were differentiated into macrophage-like cells by incubation with 50 nM phorbol 12-myristate 13-acetate (PMA; Sigma-Aldrich) for 48 h. Before stimulation of cytokine secretion, PMA-containing medium was replaced with fresh RPMI medium without PMA, and the cells were incubated for another 48 h, to allow recovery. Cell differentiation was verified by evaluation of their morphological changes under microscope. IL-6 production was induced by treating the cells with 1 μg/ml lipopolysaccharide (LPS) from *Escherichia coli* serotype O55:B5 (L6529; Sigma-Aldrich) for 24 h. IL-8 secretion was induced by exposing the cells to 25 ng/ml human recombinant IL-1β (Cell Genix, Freiburg, Germany) for 6 h. Cytokine levels were measured in the conditioned media from the stimulated cells using ELISA development kits (Mabtech), as described above. For cytokine removal experiments, the engineered bacteria (6 × 10^9^ CFU/ml) were incubated with the conditioned media from the stimulated cells or were co-cultured with the stimulated cells for 2 h. Before or after incubation with bacteria, conditioned media were collected from stimulated cells, centrifuged (5 min 2,000 g at 4°C and 15 min 1,000 g at 4°C), and stored at −80°C until analysis. The contents of the remaining cytokines in the cell culture supernatants were measured with ELISA, and the proportions (%) of cytokines removed by the engineered bacteria were calculated. Conditioned media from untreated cells were used as controls.

### Cell Transfection

HEK293 cells were seeded in 24-well plates at 2 ×10^5^ cells/well and transfected 24 h later, at approximately 70% confluency, with 1.5 µl PolyJet (SignaGen Laboratories, Rockville, MD, United States) and 0.5 µg plasmid [for expression of the EpCAM-sfGFP fusion protein, pcDNA3-EpFL-sfGFP (Gaber et al., 2018); for expression of the HER2-mEmerald fusion protein, mEmerald-ERBB2-N-18 (Addgene plasmid #62755; http://n2t.net/addgene:62755; RRID:Addgene_62755)], according to the manufacturer protocols. The medium was replaced 24 h after transfection.

### Bacterial Cell Adhesion Assay

Bacterial cell adhesion assays were carried out as reported previously (Plavec et al., 2021b). Here, the medium from transfected HEK293 cells was aspirated and 500 μl 8 × 10^8^ CFU/ml engineered *L. lactis* bacteria (diluted in pre-warmed RPMI; A_600_, 0.8) were added to each well, and incubated for 2 h at 37°C. Following incubation, the wells were gently washed twice with PBS to remove unattached *L. lactis*, fixed with 4% paraformaldehyde in PBS for 20 min at room temperature, washed twice with PBS, and mounted on a microscope slide with DAPI-containing mounting agent (Thermo Fisher Scientific), for confocal microscopy. Images were acquired with a confocal microscope (LSM-710; Carl Zeiss, Germany) using a ×63 oil-immersion objective, with settings for bright-field, DAPI (blue channel, 405 nm), GFP/mEmerald (green channel, 488 nm), and IRFP (red channel, 633 nm). Images were processed and analyzed using the ImageJ software. The adhesion of *L. lactis* cells to the HEK293 cells was quantified by counting the numbers of *L. lactis* in 10 representative microscopy images using ImageJ (Schneider et al., 2012). The results are expressed as mean numbers of *L. lactis* per human cell ±standard deviation. Human cells were counted using a cell counter plugin for ImageJ (https://imagej.nih.gov/ij/plugins/cell-counter.html), where the cells on the edges were excluded. *L. lactis* cells were counted with the particle analysis function using the Otsu threshold algorithm. To avoid background fluorescence being included as particles, the minimum size was set to 1 μm^2^. The area of a single *L. lactis* bacterium was determined by averaging areas of putative single bacteria for each set of 10 images. To determine the number of individual *L. lactis* cells, the areas of aggregates were divided by the calculated area of a single *L. lactis* bacterium.

### Statistical Analysis

Statistical analyses were performed using the Graph-Pad Prism 7.00 software (San Diego, CA, United States). All of the data are presented as means ± standard deviation (SD). The concentrations of cytokines obtained by ELISA were compared using student’s two-tailed t-tests. Differences in cytokine-binding between relevant pairs of bacteria are considered significant for *p* < 0.05.

## Results

### Design of Plasmids for Dual Protein Expression

**FIGURE 2:**
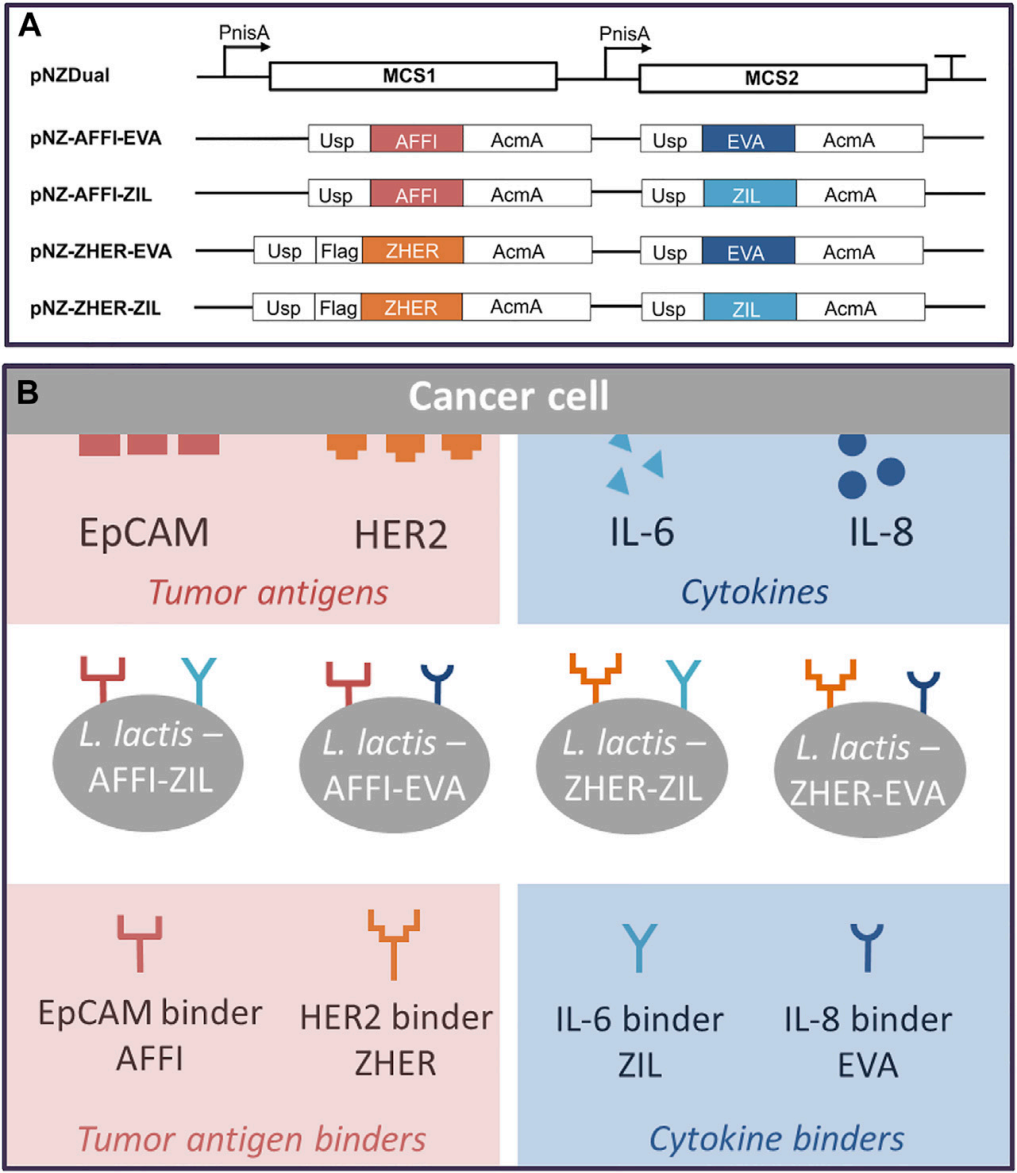
Schematic representation of the gene constructs that were cloned into the lactococcal pNZDual plasmid for co-expression and simultaneous display of tumor antigen binding proteins and cytokine binding proteins on the surface of the *Lactococcus lactis* bacteria **(A)**, with the resulting recombinant *L. lactis* strains **(B)**. AFFI, gene encoding EpCAM-binding affitin (183 bp); ZHER, gene encoding HER2-binding affibody (174 bp); EVA, gene encoding IL-8-binding evasin-3 (243 bp); ZIL, gene encoding IL-6-binding affibody (174 bp); Usp, gene encoding Usp45 signal peptide for secretion into the growth medium (84 bp); AcmA, gene encoding the C-terminal portion of the AcmA protein containing three LysM repeats for surface anchoring (642 bp); FLAG, epitope tag sequence; MCS, multiple cloning site; PnisA, nisin promoter.

Four dual plasmids containing expression cassettes for simultaneous surface display of tumor antigen binders and cytokine binders were constructed. The expression cassette of each individual binders contained the protein binder gene fused to gene for signal peptide Usp45 and the gene for the surface anchor AcmA to enable its surface display. Based on protocol described in the Materials and methods (depicted in Figure 1), tumor antigen binder gene (*zher*; *affi*) was paired with cytokine binder gene (*eva*; *zil*), resulting in dual plasmids pNZ-AFFI-EVA, pNZ-AFFI-ZIL, pNZ-ZHER-EVA, and pNZ-ZHER-ZIL (Table 1). The *L. lactis* strains transformed with the constructed dual plasmids were designated as *L. lactis*-AFFI-EVA, *L. lactis*-AFFI-ZIL, *L. lactis*-ZHER-EVA, and *L. lactis*-ZHER-ZIL. The schematic of the dual gene constructs and the resulting *L. lactis* strains are shown in Figure 2. A FLAG-tag consensus sequence was added previously between the secretion signal and the ZHER coding sequence to facilitate detection, whereas it was excluded from the AFFI fusion protein due to negative effect on its display and functionality (Plavec et al., 2021b).

### Tumor Antigen Targeting and Cytokine Binding Proteins Are Co-expressed in *L. lactis*

**FIGURE 3:**
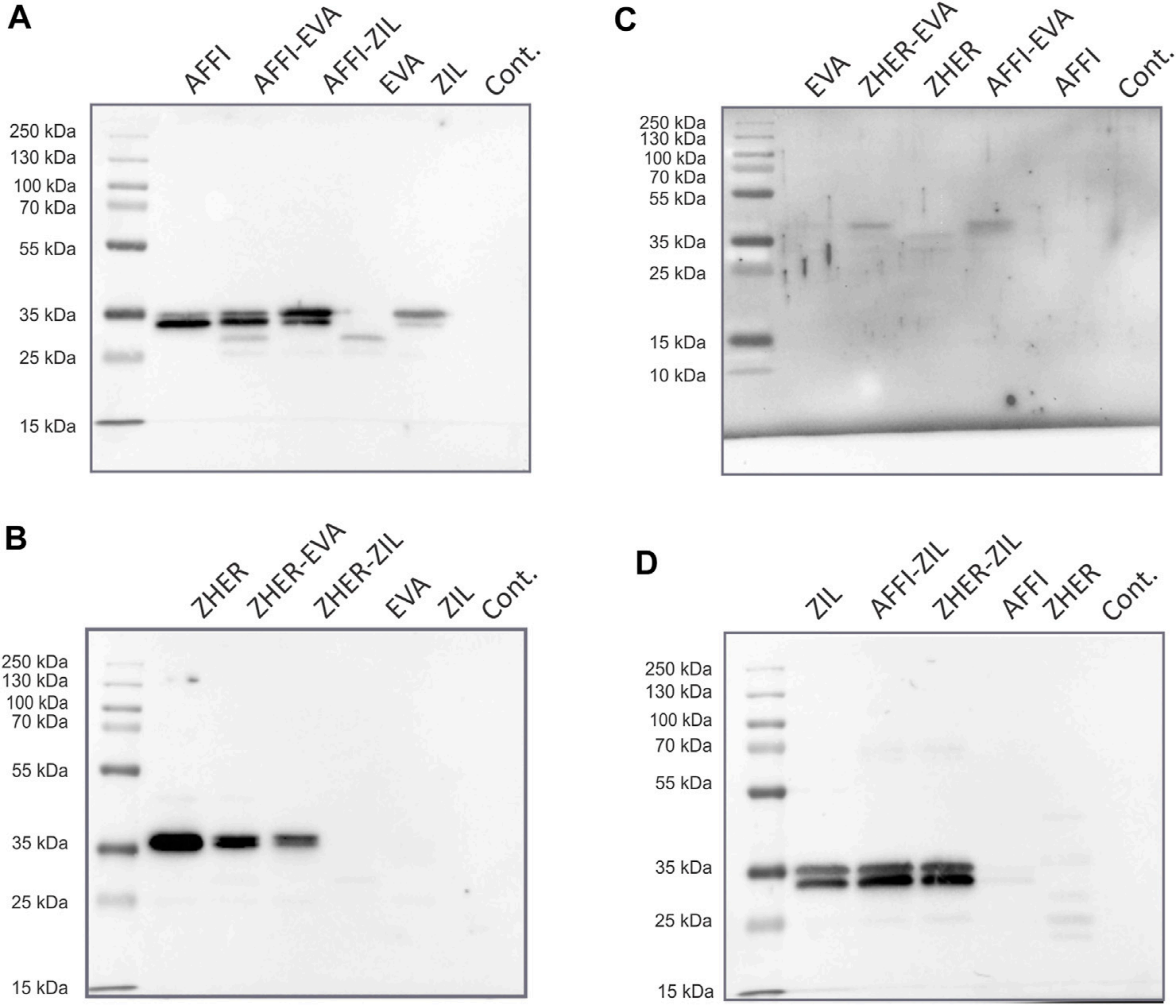
Tumor antigen targeting and cytokine binding proteins are co-expressed in *L. lactis*. Detection of tumor antigen binders AFFI **(A)** or ZHER **(B)** in combination with cytokine binders EVA **(C)** or ZIL **(D)** in the cell lysates of the dual-plasmid-containing *L. lactis* by Western blotting. Cont, *L. lactis* carrying empty plasmid pNZ8148. *L. lactis* expressing single binders (harboring pSD plasmids) served as the positive control. All binders were expressed in fusion with the Usp45 secretion signal and the AcmA surface anchor. The bands at 35 kDa correspond to the calculated molecular weights of the full-length fusion proteins (protein binder ∼7 kDa, AcmA ∼25 kDa). Recombinant human receptor EpCAM/TROP1 Fc chimera protein was used for detection of AFFI fusion protein, recombinant human receptor ErbB2/Her2 Fc chimera protein was used for detection of ZHER, recombinant human IL-8 conjugated to Fc region was used for detection of EVA, and recombinant biotinylated human IL-6 was used for detection of ZIL.

To examine the co-expression of the protein binders in the engineered *L. lactis*, whole bacterial cell lysates were analyzed by Western blotting. *L. lactis* transformed with the pSD plasmids that encoded the individual binders were used as positive controls. Immunoblotting revealed the bands at 35 kDa, which corresponds to the molecular weight of the full-length fusion proteins (protein binder ∼7 kDa, AcmA ∼25 kDa). A combination of protein binders (AFFI and EVA; AFFI and ZIL; ZHER and EVA; ZHER and ZIL) was present in the cell lysates from *L. lactis* strains carrying dual plasmids (Figure 3). Contrary to distinct bands representing binders ZHER, AFFI and ZIL, the bands representing EVA were faint or invisible for all variants, suggesting low functionality of EVA following SDS PAGE and western blot under denaturing conditions. Successful expression of EVA from pSD-EVA has been demonstrated previously (Škrlec et al., 2017) and therefore this plasmid was used in the present study as a positive control. To facilitate detection, we tagged EVA with either cmyc or flag and confirmed EVA expression in both single and dual plasmid carrying *L. lactis* strains by Western blotting using anti-cmyc and anti-flag antibodies (data not shown). The fusion proteins were consistently detected as double bands, with additional slightly higher bands that probably represent their unprocessed forms (with signal sequence). In the bacterial growth medium, only one band was identified, corresponding to the mature form of the fusion proteins (data not shown). No bands were seen for lysates of control *L. lactis* cells carrying the empty plasmid pNZ8148.

### Removal of Recombinant IL-8 and IL-6 by Engineered *L. lactis* as a Function of Bacterial Cell Number

**FIGURE 4:**
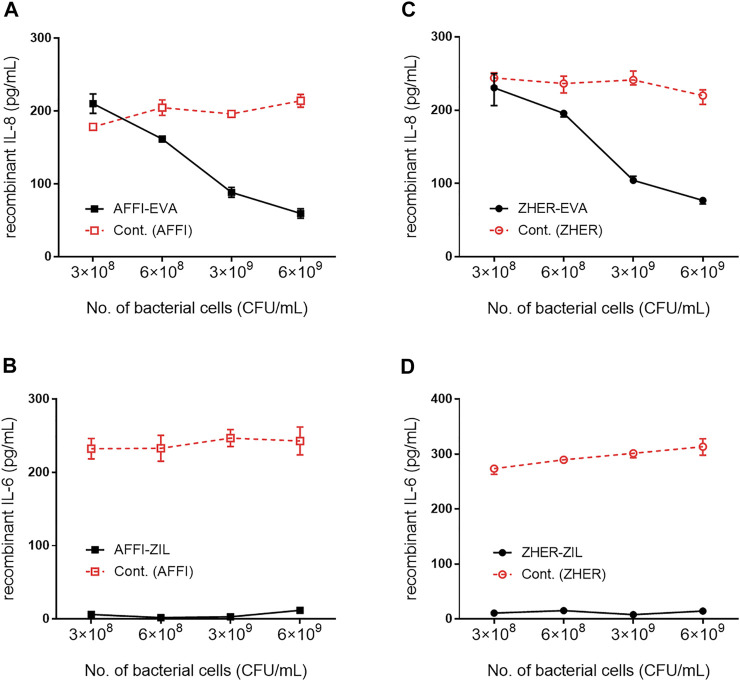
Removal of recombinant IL-8 and IL-6 by engineered *L. lactis* as a function of bacterial cell number. The concentration of recombinant IL-8 and recombinant IL-6 that remained in the solution after incubation with increasing numbers of engineered *L. lactis* cells, as determined by ELISA. Bacterial strains tested: *L. lactis* co-expressing tumor antigen targeting and cytokine binding proteins *L. lactis*-AFFI-EVA **(A)**, *L. lactis*-AFFI-ZIL **(B)**, *L. lactis*-ZHER-EVA **(C)**, or *L. lactis*-ZHER-ZIL **(D)** (solid line). Cont: *L. lactis* co-expressing tumor antigen binders and IRFP *L. lactis*-AFFI-IRFP or *L. lactis*-ZHER-IRFP (dashed line). Data are means ± SD, with experiments performed in triplicate.

The cytokine-binding ability of the engineered bacteria (i.e., *L. lactis* co-expressing tumor antigen binders and cytokine binders) was assessed by incubation of increasing numbers of bacterial cells with recombinant cytokines added to the solutions at 300 pg/ml. The levels of the cytokines that remained in the solution were determined by ELISA. The calculated recoveries were 102% for IL-8 and 98% for IL-6. The results showed that 6 ×10^9^, 3 ×10^9^, 6 ×10^8^, and 3 × 10^8^ CFU/ml *L. lactis*-AFFI-EVA removed 81, 71, 47, and 32%, respectively, of IL-8 from the solution (Figure 4A). Similarly, 6 ×10^9^, 3 ×10^9^, 6 ×10^8^ and 3 × 10^8^ CFU/ml of *L. lactis*-ZHER-EVA removed 75, 66, 36, and 25%, respectively, of IL-8 from the solution (Figure 4C). Thus, the extent of IL-8 removal correlated directly with the number of bacterial cells. In contrast, the extent of IL-6 removal was uniformly high (>95%) regardless of the number of bacterial cells tested (Figures 4B,D). The estimated minimal number of bacterial cells able to reduce a 50% of IL-8 was 10^8^ CFU/ml, whereas the minimal number of cells able to reduce a 50% of IL-6 was at least one order of magnitude lower. *L. lactis* displaying only tumor antigen binder (AFFI or ZHER) were used as the negative control and showed negligible or low binding of recombinant cytokines.

### *L. lactis* Co-Expressing IL-8 Binder EVA and the Tumor Antigen Binder AFFI or ZHER Removes IL-8 Secreted by Caco-2 and HT-29 Colorectal Cancer Cells

**FIGURE 5:**
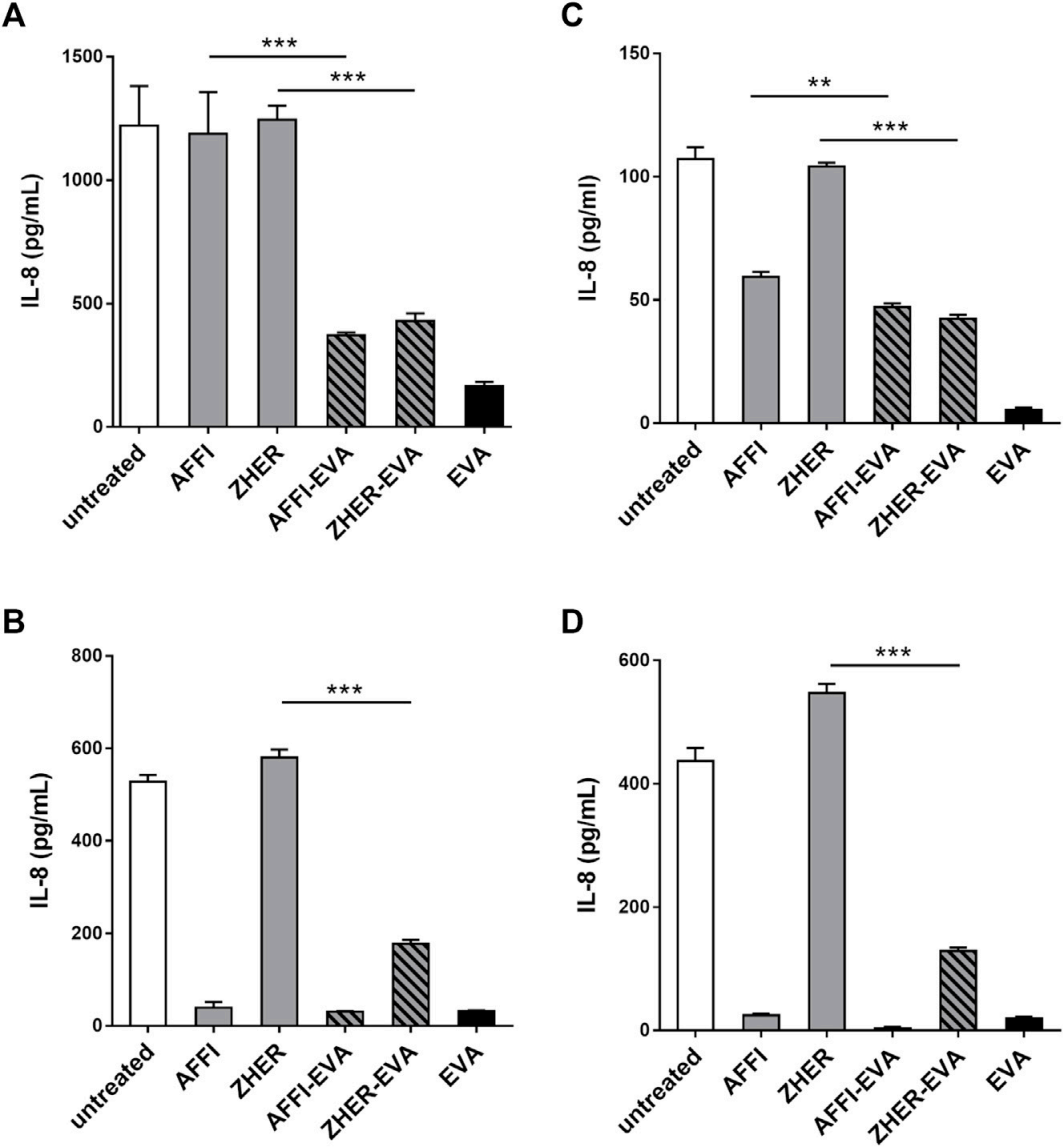
*L. lactis* co-expressing IL-8 binder EVA and the tumor antigen binder AFFI or ZHER removes IL-8 secreted by Caco-2 and HT-29 cells. ELISA-determined concentrations of IL-8 after incubation of engineered *L. lactis* (6 × 10^9^ CFU/ml) with conditioned media of IL-1β–stimulated Caco-2 cells **(A)** or HT-29 cells **(B)**, and after co-culture with IL-1β–stimulated Caco-2 cells **(C)** or HT-29 cells **(D)**. *L. lactis* strains tested: *L. lactis*-AFFI-EVA and *L. lactis*-ZHER-EVA (*L. lactis* co-expressing tumor antigen binders and EVA); Negative controls: *L. lactis*-AFFI-IRFP and *L. lactis*-ZHER-IRFP (*L. lactis* co-expressing tumor antigen binders and IRFP); Positive control: *L. lactis*-EVA (*L. lactis* expressing cytokine binder EVA only). Data are means ± SD, with experiments performed in triplicate. **, *p* = 0.001; ***, *p* < 0.001) (unpaired t-tests), between the relevant bacterial pairs (*L. lactis* co-expressing cytokine binders and tumor antigen binders, and negative controls).

The basal secretion of IL-8 into the culture medium by untreated Caco-2 and HT-29 cells was low (<10 pg/ml) or undetectable. Following 2 h treatment with IL-1β, the concentrations of IL-8 released into the supernatant of the Caco-2 and HT-29 cells ranged from 107 to 1,221 pg/ml depending on cell number or metabolic state of the cells upon stimulation (Figure 5). After incubation of engineered bacteria with IL-8–conditioned medium from IL-1β–primed cells, *L. lactis*-AFFI-EVA eliminated 65% of IL-8 from the supernatant of Caco-2 cells, and 100% of IL-8 from the supernatant of HT-29 cells. High removal efficiency of IL-8 was also achieved by *L. lactis*-ZHER-EVA, which sequestered 69% of IL-8 from the supernatant of Caco-2 cells, and 71% from the supernatant of HT-29 cells (Figures 5A,B). Furthermore, engineered *L. lactis* were shown to retain the same levels of binding when incubated in the wells along with the producer cells (Figures 5C,D). *L. lactis* expressing only EVA was included for comparison, and showed the greater cytokine removal ability in most cases (bound 17–40% more IL-8) compared with *L. lactis* strains carrying dual plasmids. As for the control bacteria, *L. lactis*-ZHER eliminated negligible amounts of IL-8. On the contrary, *L. lactis*-AFFI bound surprisingly high levels of IL-8 secreted by HT-29 cells (up to 99%), which may be due to post-translational modifications of HT-29-derived IL-8 that may have led to its non-specific binding to AFFI (Vacchini et al., 2018).

### *L. lactis* Co-Expressing IL-6 Binder ZIL and the Tumor Antigen Binder AFFI or ZHER Removes IL-6 Released by THP-1 and U-937 Monocyte-like Cells

**FIGURE 6:**
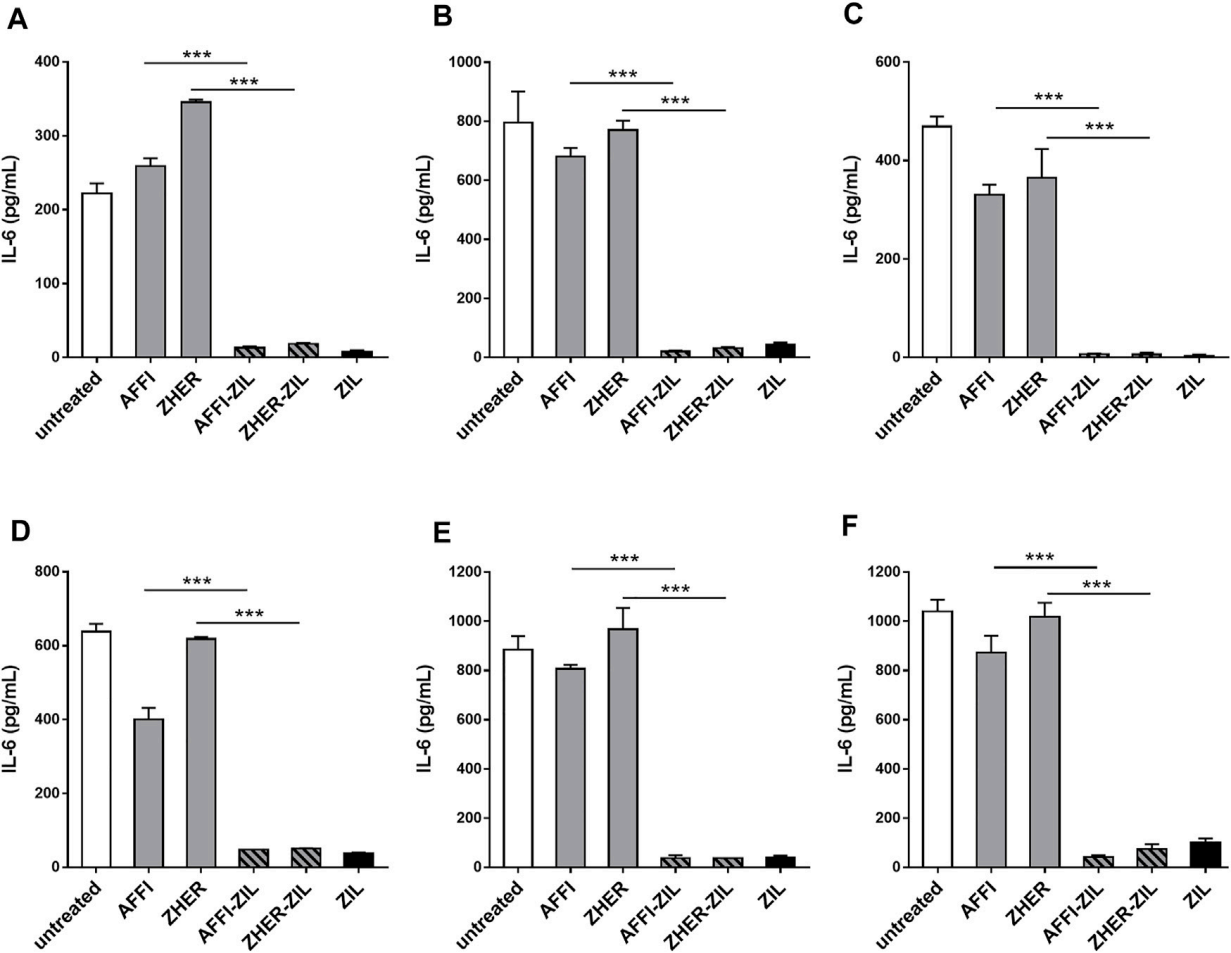
*L. lactis* co-expressing IL-6 binder ZIL and the tumor antigen binder ZHER or AFFI removes IL-6 secreted by THP-1 cells and U-937 cells. ELISA-determined concentrations of IL-6 after incubation of engineered *L. lactis* (6 × 10^9^ CFU/ml) with conditioned media of LPS-stimulated THP-1 **(A)**, differentiated THP-1 **(B)**, or differentiated U-937 **(C)** cells, and after co-culture of engineered *L. lactis* strains with LPS-stimulated THP-1 **(D)**, differentiated THP-1 **(E)**, or differentiated U-937 **(F)** cells. The cells were differentiated by incubation with phorbol 12-myristate 13-acetate for 48 h. *L. lactis* strains tested: *L. lactis*-AFFI-ZIL and *L. lactis*-ZHER-ZIL (*L. lactis* co-expressing tumor antigen binders and ZIL); Negative controls: *L. lactis*-AFFI-IRFP and *L. lactis*-ZHER-IRFP (*L. lactis* co-expressing tumor antigen binders and IRFP); Positive control: *L. lactis*- ZIL (*L. lactis* expressing cytokine binder ZIL only). Data are means ± SD, with experiments performed in triplicate. ***, *p* < 0.001 (unpaired t-tests), between the relevant bacterial pairs (*L. lactis* co-expressing cytokine binders and tumor antigen binders, and negative controls).

To assess the removal of IL-6 by the engineered bacteria, we established a cell model of inflammation by stimulating monocyte-like THP-1 cells with LPS, thus inducing IL-6 production. After LPS treatment, the cell-conditioned media were collected and the levels of secreted IL-6 were quantified by ELISA. The basal level of IL-6 was 1 pg/ml or below the detection limit. After stimulation with LPS for 24 h, the production of IL-6 increased to 222 and 638 pg/ml in two separate experiments (Figures 6A,D). For cytokine binding experiments, the engineered bacteria were incubated with IL-6–conditioned medium from LPS-stimulated cells and the residual IL-6 was determined by ELISA. As shown in Figure 6, the IL-6 binding was very efficient; *L. lactis*-AFFI-ZIL and *L. lactis*-ZHER-ZIL removed 94 and 92%, respectively, of the IL-6 from the supernatant of THP-1 cells (Figure 6A). The extent of binding was essentially equal as that of *L. lactis*-ZIL. When co-cultured with THP-1 cells, the engineered bacteria retained the same levels of IL-6 removal (Figure 6D).

In the tumor microenvironment, monocytes undergo activation and differentiation into macrophages, known as tumor-associated macrophages (Soncin et al., 2018). To examine whether the engineered bacteria can remove macrophage-derived IL-6 from their environment, we established a model of macrophages by exposing human monocytic leukemia THP-1 cells and human histiocytic lymphoma U-937 cells to PMA for 48 h. This treatment induced typical features of macrophages, such as cell adhesion, changes in morphology (from circular to spindle-shaped cells, with cellular extensions), and increased cell granularity (Schutte et al., 2009). After 24 h of stimulation with LPS, the concentration of IL-6 increased substantially. The levels of IL-6 varied in different experiments depending on the cell number, cell type (U-937 released more IL-6 than THP-1), or their differentiation status. If IL-6 concentration was above the linear range the supernatants were diluted. The supernatants that were used for incubation with bacteria contained IL-6 in the range from 469 to 1,040 pg/ml (Figures 6B,C,E,F). Similar to previous observations with undifferentiated THP-1 cells, we found that ZIL co-expressing *L. lactis* strains removed high proportion (from 96 to 99%) of the IL-6 released in the cell culture supernatant of the differentiated THP-1 cells and the differentiated U-937 cells (Figures 6B,C). When the engineered bacteria were co-cultured with the cells, the proportions of bound IL-6 remained as high as after incubation of the bacteria with conditioned media (Figures 6E,F). The control *L. lactis* strains bound a negligible amount of IL-6 (≤3%) in most cases.

### Engineered *L. lactis* Strongly and Selectively Adhere to EpCAM-Overexpressing HEK293 Cells

**FIGURE 7:**
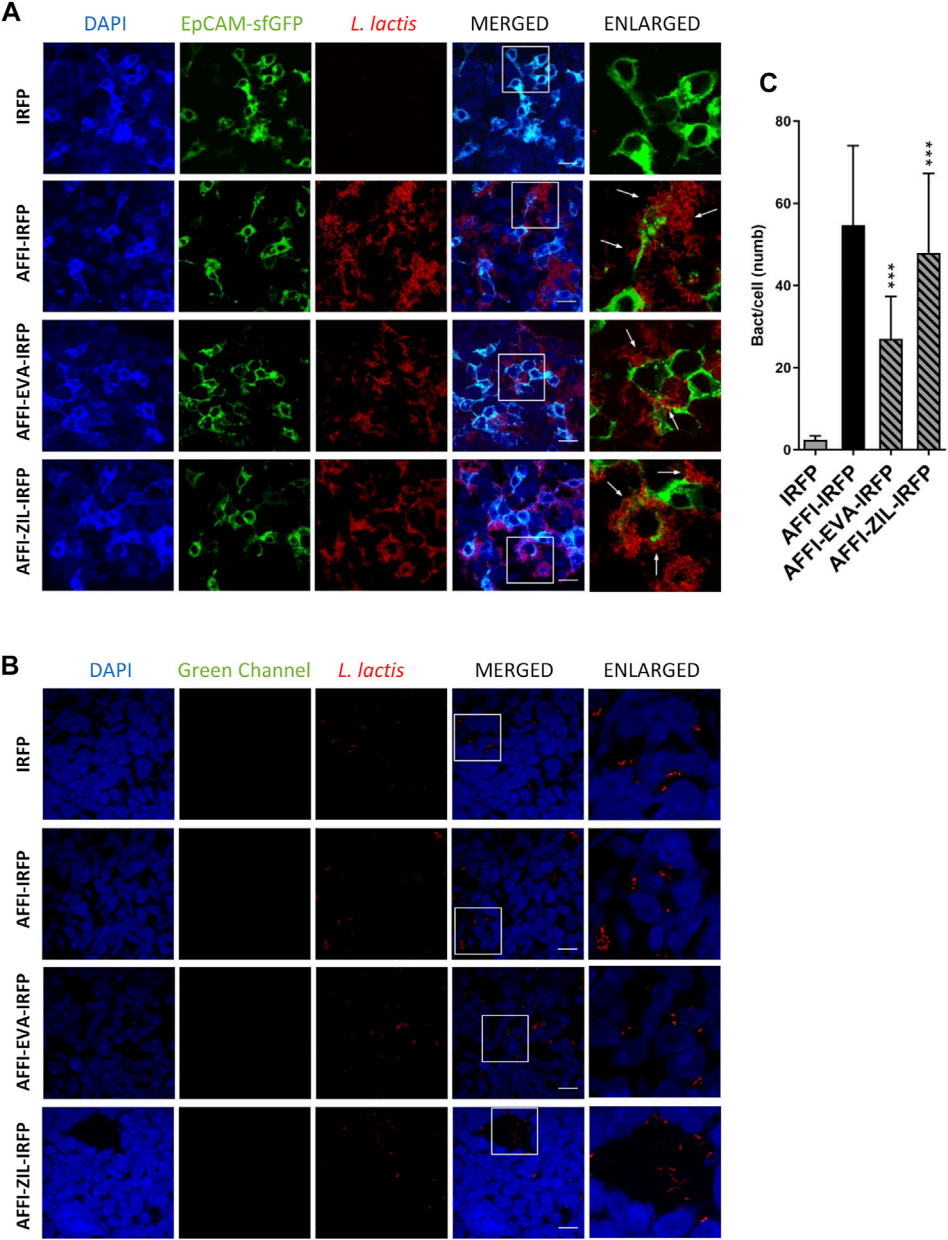
The engineered *L. lactis* strongly and selectively adheres to EpCAM-overexpressing HEK293 cells. Representative confocal microscopy images of adhesion of AFFI co-expressing *L. lactis* to HEK293 cells transfected with EpCAM **(A)** or to nontransfected HEK293 cells **(B)**. Images were quantified by determining the number of adhered bacteria per HEK293 cell in 10 representative micrographs for each experimental condition, using ImageJ **(C)**. *L. lactis* strains tested: *L. lactis*-AFFI-EVA-IRFP (*L. lactis* co-expressing AFFI, EVA, and IRFP); *L. lactis*-AFFI-ZIL-IRFP (*L. lactis* co-expressing AFFI, ZIL, and IRFP); *L. lactis*-AFFI-IRFP (*L. lactis* co-expressing AFFI and IRFP; positive control); and *L. lactis*-IRFP (*L. lactis* expressing IRFP only; negative control). DAPI, DAPI channel showing HEK293 cell nuclei; HEK293-EpCAM-sfGFP, green fluorescence channel showing superfolder green fluorescent protein (sfGFP)-labeled EpCAM overexpressed on the surfaces of the HEK293 cells; *L. lactis*, red fluorescence channel showing *L. lactis*. Enlarged images show attachment of *L. lactis* to transfected HEK293 cells with characteristic adhesion pattern (arrows). White squares indicate the enlarged sections of the images. Scale bars, 20 μm.

The feasibility of tumor targeting with AFFI co-expressing bacteria was assessed using HEK293 cells transfected with the EpCAM receptor fused to fluorescent protein superfolder GFP (sfGFP). To visualize bacterial adhesion by fluorescence microscopy, we used *L. lactis* that co-expressed the IRFP together with the tumor antigen binders and cytokine binders (Plavec et al., 2021a). Surface presentation of the protein binders and intracellular IRFP expression were confirmed by flow cytometry and fluorescence measurements, respectively (Plavec et al., 2021a). Confocal microscopy showed that the engineered bacteria *L. lactis*-AFFI-EVA-IRFP and *L. lactis*-AFFI-ZIL-IRFP strongly and selectively adhered to EpCAM-overexpressing HEK293 cells, in contrast to the control strain *L. lactis-*IRFP, which showed no adhesion ability (Figure 7A). Particular adhesion pattern was observed with the clustering of the bacteria at the cell edges (Figure 7A, arrows). On average, 27 *L. lactis*-AFFI-EVA-IRFP, 48 *L. lactis*-AFFI-ZIL-IRFP, and 55 *L. lactis*-AFFI-IRFP (positive control) adhered per single EpCAM-bearing HEK293 cell, whereas only 2 *L. lactis*-IRFP (negative control) adhered per single EpCAM-bearing HEK293 cell (Figure 7C). Compared to the positive control, a 49% and a 13% reduction of the adhesion was noticed for *L. lactis*-AFFI-EVA-IRFP and *L. lactis*-AFFI-ZIL-IRFP, respectively. The average number of adhered bacteria per nontransfected HEK293 cell was <1; hence, the AFFI co-expressing bacteria showed no reactivity toward nontransfected cells (Figure 7B). These data confirm that the displayed EpCAM-targeting tumor antigen binder AFFI effectively and specifically promotes adherence of *L. lactis* to the transfected EpCAM-bearing HEK293 cells.

### Engineered *L. lactis* Selectively Adhere to HER2-Overexpressing HEK293 Cells

**FIGURE 8:**
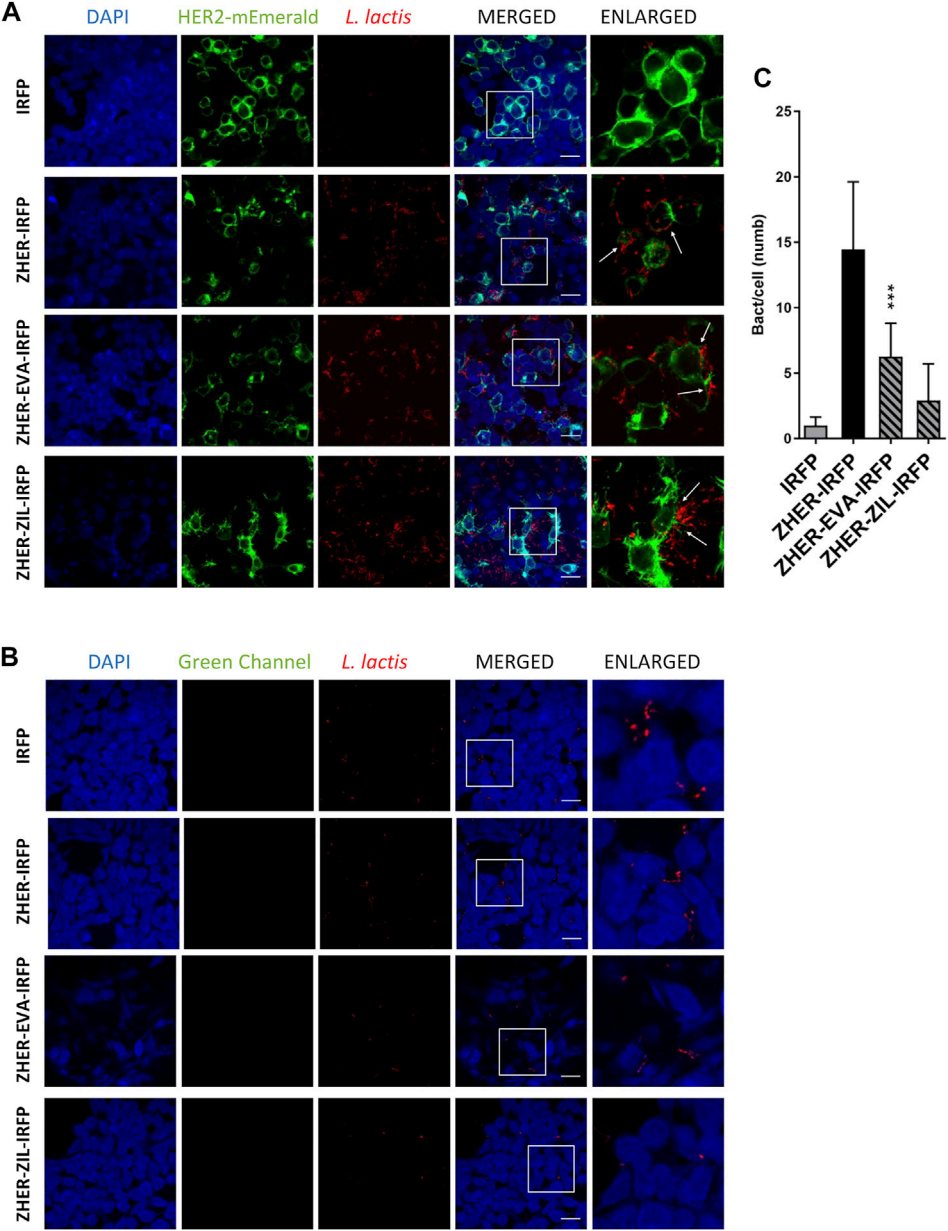
The engineered *L. lactis* specifically adhere to HER2-overexpressing HEK293 cells. Representative confocal microscopy images of adhesion of ZHER co-expressing *L. lactis* to HEK293 cells transfected with HER2 **(A)** or to nontransfected HEK293 cells **(B)**. Images were quantified by determining the number of adhered bacteria per HEK293 cell in 10 representative micrographs for each experimental condition, using ImageJ **(C)**. *L. lactis* strains tested: *L. lactis*-ZHER-EVA-IRFP (*L. lactis* co-expressing ZHER, EVA, and IRFP); *L. lactis*-ZHER-ZIL-IRFP (*L. lactis* co-expressing ZHER, ZIL, and IRFP); *L. lactis*-ZHER-IRFP (*L. lactis* co-expressing ZHER and IRFP; positive control); and *L. lactis*-IRFP (*L. lactis* expressing only IRFP; negative control). DAPI, DAPI channel showing HEK293 cell nuclei; HEK293-HER2-mEmerald, green fluorescence channel showing mEmerald-labeled HER2 overexpressed on the surfaces of the HEK293 cells; *L. lactis*, red fluorescence channel showing *L. lactis*. Enlarged images show attachment of *L. lactis* to transfected HEK293 with characteristic adhesion pattern (arrows). White squares indicate enlarged sections of the images. Scale bars, 20 μm.

The feasibility of tumor targeting with ZHER co-expressing bacteria was investigated using HEK293 cells transfected with HER2 receptor fused to fluorescent protein monovalent variant of GFP mEmerald. The attached bacteria were visualized by confocal fluorescence microscopy *via* the IRFP reporter protein that was co-expressed in *L. lactis* with the protein binders (Plavec et al., 2021a). Confocal microscopy showed that the engineered bacteria selectively adhered to HER2-overexpressing HEK293 cells and showed no reactivity towards nontransfected HEK293 cells (Figures 8A,B). The adhesion pattern of the HER2-targeting bacteria was similar to that of the EpCAM-targeting *L. lactis* strains, with the bacteria mostly attached to the cell edges (Figure 8A, arrows). Quantification showed that on average 6 *L. lactis*-ZHER-EVA-IRFP, 3 *L. lactis*-ZHER-ZIL-IRFP, 14 *L. lactis-*ZHER-IRFP (positive control), and 1 *L. lactis-*IRFP (negative control) adhered per HER2-bearing HEK293 cell (Figure 8C). Compared with positive control, a 57% and a 79% reduction of the adhesion was noticed for *L. lactis*-ZHER-EVA-IRFP and *L. lactis*-ZHER-ZIL-IRFP, respectively. ZHER co-expressing bacteria showed no reactivity towards nontransfected HEK293 cells, with no more than 1 bacterium adhering per nontransfected HEK293 cell (Figure 8B). These data confirm that the displayed HER2-targeting tumor antigen binder ZHER effectively and specifically promotes adherence of *L. lactis* to the transfected HER2-bearing HEK293 cells. However, the adhesion level was significantly lower for the HER2-targeting *L. lactis* strains compared with the EpCAM-targeting *L. lactis* strains.

## Discussion

Tumorigenesis in CRC is driven by soluble mediators, including the pro-inflammatory cytokines IL-6 and IL-8, which orchestrate the many-fold cellular activities that underlie inflammation. Consequently, modulation of these cytokines might be a promising therapeutic strategy for cancer treatments. Here, we engineered *L. lactis* to serve as a vector for targeted delivery of cytokine-binding proteins to tumor. The *L. lactis* bacteria were constructed by displaying a tumor antigen-recognizing moiety and a cytokine-binding moiety on their surface. These engineered dual functionalized bacteria were shown to selectively target HEK293 cells that overexpress tumor antigens. Moreover, the engineered bacteria removed a high proportion of the IL-8 from the supernatants of immunostimulated Caco-2 and HT-29 colon adenocarcinoma cell lines, and depleted IL-6 from the supernatants of immunostimulated THP-1 and U-937 monocyte-like cells.

*L. lactis* with dual functionality was developed by simultaneously displaying a combination of a tumor antigen-recognizing protein and a cytokine-binding protein on the bacterial surface using a dual promoter plasmid (pNZDual). IL-8–binding evasin (designated as EVA) and IL-6–binding affibody (designated as ZIL) were used to achieve cytokine removal, while EpCAM-binding affitin (designated as AFFI) and HER2-binding affibody (designated as ZHER) served for tumor antigen targeting.

After confirming the co-expression of the fusion proteins in all dual plasmid carrying *L. lactis* strains, their cytokine binding properties were investigated in relation to the bacterial cell number using recombinant cytokines. The engineered bacteria removed more than 90% of recombinant IL-6 from the solution independent of bacterial cell numbers tested, whereas they removed between 25 and 81% of recombinant IL-8 in a dose-dependent manner. In agreement with previous study (Škrlec et al., 2017), the highest IL-8 removal was achieved by 6 × 10^9^ CFU/ml bacterial cells, which falls within the range of therapeutic doses in current probiotic preparations for human use. Although typical doses of probiotics vary by product, most clinical studies have examined doses from 1 to 20 billion CFU per day, with higher doses generally associated with better therapeutic outcomes (i.e., >10 billion CFU per day in adults) (Kligler and Cohrssen, 2008). Therefore, we chose 6 × 10^9^ CFU/ml for testing removal of IL-8 and IL-6 from the cell culture supernatants.

The inflammatory cycle in cancer is driven by the pro-inflammatory cytokines that are released in the tumor microenvironment by cancer cells, in concert with immune cells. To assess the removal of the cytokines involved in cancer inflammatory processes by the engineered bacteria, we established cell models that mimic certain elements of the inflammatory milieu within the tumor microenvironment. Under inflammatory conditions, cancer cells are a source of IL-8 in the tumor microenvironment. We used the Caco-2 and HT-29 cell lines as a model of CRC cells. To simulate inflammatory conditions, the Caco-2 and HT-29 CRC cells were primed with IL-1β, a potent inducer of IL-8 secretion. The optimal dose of IL-1β required to induce maximal IL-8 secretion in Caco-2 cells was determined to be 25 ng/ml (Škrlec et al., 2017). Autocrine secretion of IL-8 can activate intrinsic mechanism of tumor cells to promote their escape from stress-induced apoptosis (Doll et al., 2010). Cancer-cell-derived IL-8 can enhance tumor growth, invasion, angiogenesis, metastasis, and resistance to therapy (Doll et al., 2010). In breast cancer, it has been shown that expression of IL-8 can be modulated by the manipulation of EpCAM expression *in vitro* (Sankpal et al., 2013). Here, we show that cell-released IL-8 can be effectively captured and removed by the engineered bacteria both in the presence and absence of the producer cells. IL-8 removal was substantial, whereby *L. lactis* co-expressing EVA and tumor antigen binders removed 65–69% of IL-8 from Caco-2 supernatant and 71–100% of IL-8 from supernatant of HT-29 cells.

In addition to cancer-cell-derived mediators, pro-inflammatory cytokines produced by immune cells (i.e., primarily monocytes and macrophages) also drive inflammation in the CRC microenvironment (Hao et al., 2012). Monocytes are attracted to tumor tissue by chemokines such as IL-8, which is one of the major chemoattractants in CRC (Ning et al., 2011). In the tumor microenvironment, monocytes respond to paracrine stimuli from cancer cells by secreting soluble mediators, most notably IL-6, and these participate in tumor growth, invasion, intravasation, and metastasis (Soncin et al., 2018). For the analysis of IL-6 removal by the engineered bacteria, we used the immunostimulated THP-1 human acute monocytic leukemia cell line and the U937 histiocytic lymphoma cell line. PMA-differentiated THP-1 and U-937 cells were used as cell models of macrophages. It is known that the production of pro-inflammatory cytokines increases when monocytes differentiate into macrophages, which suggests that they are more likely to induce inflammation than monocytes (Soncin et al., 2018). After incubation of the engineered bacteria with stimulated cells or their conditioned medium, high levels of IL-6 were removed (>90%) by ZIL co-expressing *L. lactis* in both the undifferentiated and differentiated monocyte-like cell lines. The removal of IL-6 from cell culture supernatants by the dual functionalized bacteria was comparable to that by its single binder counterpart (*L. lactis* that expressed only ZIL). This confirms that IL-6 removal by the engineered bacteria was not affected by the presence of tumor antigen binders on their surface. Notably, IL-6 removal by the ZIL co-expressing bacteria was more pronounced than IL-8 removal by the EVA co-expressing bacteria, even though both binders have similar affinity constants (Kd ∼500 pM for ZIL-6 (31), Kd ∼430 pM for EVA (Deruaz et al., 2008)). This finding highlights that in addition to the affinities, the interactions of the ligand-displaying bacteria with the target depend on the extent of ligand display, its surface accessibility and/or spatial orientation. Overall, the cytokine removal by the engineered bacteria was high and effective across a range of cytokine concentrations (107–1,040 pg/ml). Thus, the cytokine binders expressed on *L. lactis* remained biologically active and bound the IL-8 and IL-6 in the media of four different cell lines.

Previous studies have shown that neutralization of IL-8 using monoclonal antibodies or small-interfering RNAs is not sufficient to block signaling of other chemokines in the tumor microenvironment (Ning and Lenz, 2012). Moreover, disruption of a single cytokine might trigger compensatory mechanisms that maintain pro-tumorigenic inflammation and immunosuppression (Jones et al., 2016). These studies suggest that the agent designed to attenuate the effect of a single cytokine involved in cancer inflammation is not sufficient for a complete clinical effect. Here, we have shown that multiple cytokines can be targeted by engineered *L. lactis*. The developed bacteria can be further tested for their ability to achieve additive or synergistic effects as a means to overcome cytokine redundancy.

Bacteria have a natural propensity to colonize tumor, and although they have been administered safely in clinical trials for cancer treatment (Toso et al., 2002), efficient tumor colonization requires administration of high bacterial number into the bloodstream and therefore has a narrow therapeutic index. The oral administration route is a path to selectively deliver bacteria to the CRC tumor microenvironment while minimizing systemic exposure. Oral delivery of nonpathogenic probiotic strains equipped with tumor antigen ligands for specific tumor targeting might lead to preferential accumulation of bacteria in the CRC as it allows bacteria to closely interact with tumor tissue, thus increasing drug availability in close proximity to responsive cells and limiting dilution into the luminal fluid.

The interactions between the engineered bacteria and HEK293 cells that overexpress tumor antigens EpCAM or HER-2 were investigated by fluorescent staining and confocal microscopy. We found that the engineered bacteria selectively adhered to the transfected cells, with a high level of adhesion observed for AFFI co-expressing bacteria, and a moderate level of adhesion for ZHER co-expressing bacteria. The ligand-receptor interaction is generally influenced by the affinity of the ligand for the receptor, the expression and surface accessibility of the receptor, and the rate of receptor internalization upon ligand binding. The difference in relative adhesion efficiency between AFFI and ZHER expressing strains probably reflects the lower surface accessibility of HER2 receptor compared to EpCAM rather than the affinity, considering that ZHER has a higher affinity constant (Kd = 22 pM) for HER2 than AFFI for EpCAM (Kd = 110 pM). The interactions were highly specific as nontargeted bacteria (i.e., *L. lactis* expressing only IRFP intracellularly) showed no adhesion activity towards transfected HEK-293 cells, and neither *L. lactis* strain adhered to nontransfected cells. Hence, by displaying tumor antigen binding proteins on their surface, *L. lactis* with strong cell adhesion properties were developed from a nonadhesive *L. lactis* strain. This means that the adhesion is achieved *via* the interaction between the tumor antigen ligand on the bacteria and the receptor on the cell surface, rather than *via* the nonspecific interactions between the peptidoglycans on the bacteria and the cell surface.

In general, the microscopy revealed that all of the *L. lactis* strains tested were attached to the cell edges. Such an adhesion pattern appears to be characteristic of *L. lactis*, and is consistent with previous reports of dense clusters of *L. lactis* IBB477 strain interacting with mucus secreted by HT29-MTX cells (Radziwill-Bienkowska et al., 2017). Similarly, previous studies have shown that *Lactobacillus* strains also preferentially adhere to cell edges, with the exception of highly adhesive *Lb. plantarum* C9S2, which adhered throughout the monolayer of the NCM460 normal human colon mucosal epithelial cell line (Garcia-Gonzalez et al., 2018).

Overall, we have demonstrated here that engineered bacteria can completely remove or markedly reduce the concentrations of IL-8 and IL-6 in the media of four different cell lines. Furthermore, these engineered bacteria exerted tumor antigen targeting in cell cultures, whereby strong and highly specific adhesion to HEK293 cells overexpressing the tumor antigens EpCAM or HER2 was achieved, particularly by the EpCAM-targeting *L. lactis* strains. When dual functionalized bacteria were compared with *L. lactis* strains that display only cytokine binders, the EVA co-expressing dual *L. lactis* strains removed less IL-8, whereas the ZIL co-expressing dual *L. lactis* strains removed the same amount of the IL-6 and no effect of tumor antigen binders was observed. As expected, the adhesion of dual *L. lactis* strains was lower (about a 10% to a 80% reduction) compared to the *L. lactis* displaying the tumor antigen binders only. Of the four *L. lactis* strains tested, *L. lactis*-AFFI-ZIL-IRFP showed the highest cytokine removal and adhesion efficiency, thus showing the greatest potential for further exploration of cytokine-targeted therapeutic strategies against cancer.

## Conclusion

In this study, we have developed a set of new *L. lactis* strains with dual functionality by displaying two distinct bioactive moieties on the bacterial surface. The surface display of EpCAM- or HER2-binding proteins enabled selective targeting of the cells overexpressing tumor antigens, while the attachment of the IL-8- or IL-6-binding proteins to the *L. lactis* surface provided the bacteria with cytokine removal capacity. To the best of our knowledge, this is the first report of recombinant *L. lactis* equipped with ligands for tumor antigen targeting and cytokine neutralization. By using other therapeutic and/or diagnostic domains that bear unique functionalities, this strategy can be readily applied to target other relevant molecules involved in the pathogenesis of cancer or other diseases.

## Data Availability

The original contributions presented in the study are included in the article/supplementary material, further inquiries can be directed to the corresponding author.

## Abbreviations

CFU, colony-forming-unit; CRC, colorectal cancer; EpCAM, epithelial cell adhesion molecule; HER2, human epidermal growth factor receptor 2; HRP, horseradish peroxidase; IL, interleukin; IRFP, infrared fluorescent protein; MCS, multiple cloning site; PBS, phosphate-buffered saline; PMA, phorbol 12-myristate 13-acetate; TBS, Tris-buffered saline.
